# Impact of public health team engagement in alcohol licensing on health and crime outcomes in England and Scotland: A comparative timeseries study between 2012 and 2019

**DOI:** 10.1016/j.lanepe.2022.100450

**Published:** 2022-06-30

**Authors:** Frank de Vocht, Cheryl McQuire, Claire Ferraro, Philippa Williams, Madeleine Henney, Colin Angus, Matt Egan, Andrea Mohan, Richard Purves, Nason Maani, Niamh Shortt, Laura Mahon, Gemma Crompton, Rachel O'Donnell, James Nicholls, Linda Bauld, Niamh Fitzgerald

**Affiliations:** a Population Health Sciences, Bristol Medical School, University of Bristol, UK; b NIHR School for Public Health Research, UK; c NIHR Applied Research Collaboration West, UK; d School of Health and Related Research, University of Sheffield, UK; e SPECTRUM Consortium, UK; f Department of Public Health, Environments and Society, London School of Hygiene & Tropical Medicine, UK; g School of Health Sciences, University of Dundee, UK; h Institute for Social Marketing & Health, University of Stirling, UK; i Boston University School of Public Health, USA; j School of GeoSciences, University of Edinburgh, UK; k Alcohol Focus Scotland, UK; l Usher Institute, University of Edinburgh, UK

**Keywords:** Alcohol, Public health, Alcohol licensing, Crimes, Hospital admissions, Ambulance, Timeseries, Growth models, Policy

## Abstract

**Background:**

Public health teams (PHTs) in England and Scotland engage to varying degrees in local alcohol licensing systems to try to reduce alcohol-related harms. No previous quantitative evidence is available on the effectiveness of this engagement. We aimed to quantify the effects of PHT engagement in alcohol licensing on selected health and crime outcomes.

**Methods:**

39 PHTs in England (*n* = 27) and Scotland (*n* = 12) were recruited (of 40 contacted) for diversity in licensing engagement level and region, with higher activity areas matched to lower activity areas. Each PHT's engagement in licensing for each 6 month period from April 2012 to March 2019 was quantified using a new measure (PHIAL) developed using structured interviews, documentary analyses, and expert consultation. Outcomes examined were ambulance callouts, alcohol-related hospital admissions, alcohol-related and alcohol-specific mortality and violent, sexual and public order offences. Timeseries were analysed using multivariable negative binomial mixed-effects models. Correlations were assessed between each outcome and 18-month average PHIAL score (primary metric), cumulative PHIAL scores and change in PHIAL scores. Additionally, 6-month lagged correlations were also assessed.

**Findings:**

There was no clear evidence of any associations between the primary exposure metric and the public health or crime outcomes examined, nor between cumulative PHIAL scores or change in PHIAL score and any outcomes. There were no significant associations in England or Scotland when analysed separately or between outcomes and lagged exposure metrics.

**Interpretation:**

There is no clear evidence that allocating PHT resources to engaging in alcohol licensing is associated with downstream reductions in alcohol-related health harms or crimes, in the short term or over a seven year follow-up period. Such engagement likely has benefits in shaping the licensing system to take account of health issues longer term, but as current systems cannot reduce alcohol availability or contain online sales, their potential benefits are somewhat constrained.

**Funding:**

The ExILEnS project is funded by the NIHR Public Health Research Programme (project number 15/129/11). The views expressed are those of the authors and not necessarily those of the NHS, the NIHR or the Department of Health.

**Research in context**

**Evidence before this study**

Alcohol consumption is a major contributor to the preventable burden of disease and crime globally. In England and Scotland, a licence issued by a local government ‘licensing committee’ is required to legally sell alcohol. Such committees must operate to statutory licensing objectives and produce and consult on a local statement of licensing policy which outlines their strategic approach. Various stakeholders, including public health teams (PHTs), have statutory roles in the licensing system and seek to influence licensing policy and decisions in diverse ways, committing time and resources. The impact of such PHT engagement has not previously been examined.

**Added value of this study**

This study finds that greater PHT engagement in alcohol licensing is not associated with measurably lower hospital admissions or crimes related to alcohol misuse, or overall ambulance call-outs, in the short term, nor over the 7-year follow-up period of this study. Findings were similar when examining the data for Scotland only, where a public health objective for licensing exists, and for England only, where it does not.

**Implications of all the available evidence**

Complementary qualitative research from the ExILEnS project suggests that the allocation of PHT resources to engaging in alcohol licensing may be slowly re-orienting licensing systems, policies and committees to take account of health considerations (especially in Scotland), and is valued by licensing stakeholders. Our findings suggest that it is unrealistic to expect such engagement to result in measurable improvements in health or crime in the medium term, most likely because the licensing systems as currently designed, cannot reduce availability of alcohol, nor contain online sales.

Alt-text: Unlabelled box

## Introduction

Alcohol consumption is a major contributor to the preventable burden of disease in the UK and internationally.^1^^,^^2^ The UK Office for National Statistics reported 7565 alcohol-specific deaths in the UK in 2019, corresponding to an age-standardised death rate of 11.8 per 100,000 people: at that time, the second highest tally since their data time series began in 2001.^3^ Alcohol harms are socially patterned, making alcohol a key driver of health inequalities.^4^

There is consistent evidence of an association between increased physical and temporal availability of alcohol, higher rates of consumption, and associated alcohol-related harms.^5^ The mechanisms by which effects occur however, remain unclear and likely differ depending on contexts.^6^ In England and Scotland, as with other jurisdictions in the UK and many other countries, the sale of alcohol requires a licence. Licences are issued by local government (LG) bodies known as Licensing Authorities (England) or Boards (Scotland). The licensing systems in both nations was extensively revised in national legislation from the early 2000s onwards to introduce licensing objectives, set formal roles for stakeholders, and require local licensing policies to be developed. Firstly, statutory ‘licensing objectives’ were established to guide decisions to grant, amend or refuse licence applications: In England and Scotland these objectives include preventing crime and disorder, promoting public safety, preventing public nuisance, and protecting children from harm, while Scotland has the additional objective of protecting and improving public health. Secondly, several bodies were specified in law as ‘responsible authorities’ (England) or ‘statutory consultees’ (Scotland) who must be notified of licence applications and can seek to influence licensing decisions, including by making a representations on applications. English health authorities became classed as responsible authorities in 2012 and, in 2013, many public health functions in England were transferred from the NHS to Local Government.^7^ As a result, LG-based public health practitioners gained a statutory role in the licensing system. In Scotland, public health professionals based in local NHS Boards became statutory consultees from 2011.

In addition to considering individual applications, Licensing Authorities/Boards are required to produce and consult on a local ‘statement of licensing policy’ (SLP), which outlines their strategic approach to promoting the licensing objectives, and to which health and other stakeholders can input. Recent studies reported associations between the intensity of active licensing policies in LG areas in England and reductions in alcohol-related hospital admissions,^8^ public nuisance offences, and alcohol-related crimes.^9^^,^^10^ Research into the role of public health stakeholders in this context suggests that the lack of a public health objective for licensing in England could undermine public health influence in local licensing systems.^7^

Many Public Health Teams’ (PHT) have committed resources to engaging in licensing matters,^11^ notwithstanding fiscal restrictions on LG over the last decade and continued financial uncertainty affecting public health and other budgets.^12^ The “Exploring the Impact of alcohol premises Licensing in England and Scotland” (ExILEnS) study aimed to critically assess the impact and mechanisms of impact of public health stakeholders’ engagement in alcohol premises licensing from 2012 to 2019 on local alcohol-related harms.^13^ This paper describes the ExiLEnS quantitative evaluation of whether the intensity of public health engagement in alcohol licensing is associated with changes in alcohol-related harms.

## Methods

We employed multivariable timeseries analyses of LG areas using a semi-quantitative metric of PHT activity in alcohol licensing. The protocol of the ExILEnS study was previously published,^13^ and the analytic plan was registered prior to the start of analyses (Version 1.02 uploaded on 23.10.2020; https://osf.io/ag8qn/).

### Population

The recruitment of PHTs in 39 LG areas (27 in England, 12 in Scotland) was described in our protocol^13^ and in detail elsewhere.^14^ In the absence of quantitative data on effects of PHT engagement in licensing on harms, sample size was informed by statistical power calculations based on effect sizes of the effect of alcohol premises licensing on alcohol-related hospital admissions and reported crime rates.^9^^,^^15^ In short, 20 PHTs from different regions and varying levels of rurality in Scotland and England determined *a priori* to have PHTs actively engaged in alcohol licensing were recruited. To maximise sample exposure variation, these were matched to 20 other LG areas with PHTs less active in licensing. Matching was done using ‘optimal’ propensity score matching (*MatchIt* 4.2.0 package in R 4.0.5) in England and directly for Scottish areas because of the limited number of areas in Scotland. Matching variables were year 2009 area-level deprivation, population density, on-licence and off-licence outlet densities ('population-weighted mean outlet density based on a 1 km radius), alcohol-related hospital admissions, alcohol-related crimes, rurality and median age. Where the PHT in a matched area declined to participate, or was found to be highly active in engagement with licensing, they were excluded and matching was re-run. One recruited matched area dropped out, leaving 39 final participating areas whose characteristics are described in^14^ and Online Supplement Materials (OSM) Table S1.

### Exposure

Through structured interviews and documentary analysis, local PHT's engagement in alcohol licensing was examined from April 2012 to March 2019 for 19 activity types organised into 6 categories (OSM Table S2). Activity levels were assessed, graded, and combined in a semi-quantitative composite measure (the PHIAL measure), to give a score for each 6-month period, ranging from 0 if no engagement during the period to a theoretical maximum of 42. Full details of the definition, grading scales and weighting of each activity type are described elsewhere.^14^ Based on discussions within the research team and with external experts and practitioners, the primary exposure metric was average PHT activity over the preceding 18 months, which was thought of as a plausible period by which any effects might occur. Secondary exposure metrics were cumulative PHT activity score to date – to explore the impact of the level of PHT activity over a prolonged period, and the change in the composite score at each timepoint – to explore immediate changes in alcohol harm outcomes arising from changes in engagement. For all metrics an additional 6-month lag between exposure and outcomes, as well as nation-specific associations, were also modelled. Additional *posthoc* analyses not included in the protocol examined extended lags of 12 and 18 months for all metrics.

### Outcomes

Alcohol harm outcomes were selected *a priori,* aggregated to 6-month periods, and include alcohol-related hospital admissions (narrow measure - the primary outcome), hospital admissions for acute conditions related to alcohol, alcohol-related mortality, alcohol-specific mortality, all ambulance callouts, and sexual, violent and public order offences. More information, including sources of data and specifics of data management, is provided in OSM Table S3. In total this resulted in a dataset of 546 observations (39 areas with 14 timepoints) for all outcomes, with the exception of ambulance callouts (*n* = 532). There was no missing outcome data. In a deviation from protocol we did not include A&E attendances for alcohol because of difficulties with overlap between A&E capture areas and study areas.

### Covariates

Covariates considered potential confounding factors for modelling were nation (England or Scotland), season, area-level socio-economic status (Index of Multiple Deprivation), population density, average age, baseline PHIAL score and baseline outcome, and, for England only, whether an area was a local alcohol action area (LAAA). LAAAs were a Home Office initiative in which local agencies, including licensing authorities, health bodies, the police, and businesses and other organisations work together to address alcohol-related problems.^16^^,^^17^

### Statistical modelling

Time series of outcome incidents for each 6-month period were modelled using negative binomial mixed effects models incorporating a random intercept for each area. Mid-year population size was used as an offset. Non-linearity was assessed by inclusion of 2nd-degree polynomial terms, but this did not significantly improve model fit for any outcome. Nation-specific trends were incorporated where they improved model fit. Associations between each outcome and 18-month average PHIAL, ‘cumulative PHIAL score’ and ‘change in PHIAL score’ were modelled as ‘snares’, in which the PHIAL score at each time point was hypothesized to impact on the outcome in the same 6-month period (OSM Figure S1) or as 6-month lagged snare models (OSM Figure S2). Baseline models included PHIAL score, time, population size offset and random intercept for each area. Fully adjusted models were developed, but, for reporting, the most parsimonious models were selected based on AIC values and stability of predictors. At this level of geographical aggregation spatial dependence was thought to be minimal, and this was not included in any models. Where statistically significant associations (*p* < 0.05) were observed, 1-holdout resampling was conducted in which areas were sequentially removed and models recalculated. All analyses were undertaken using the *glmer.nb* procedure (*lme4* package) in *R* (version 4.05).

### Role of funding source

The funders had no role in study design, data collection, data analysis, interpretation, or in writing the report.

## Results

**Figure 1:**
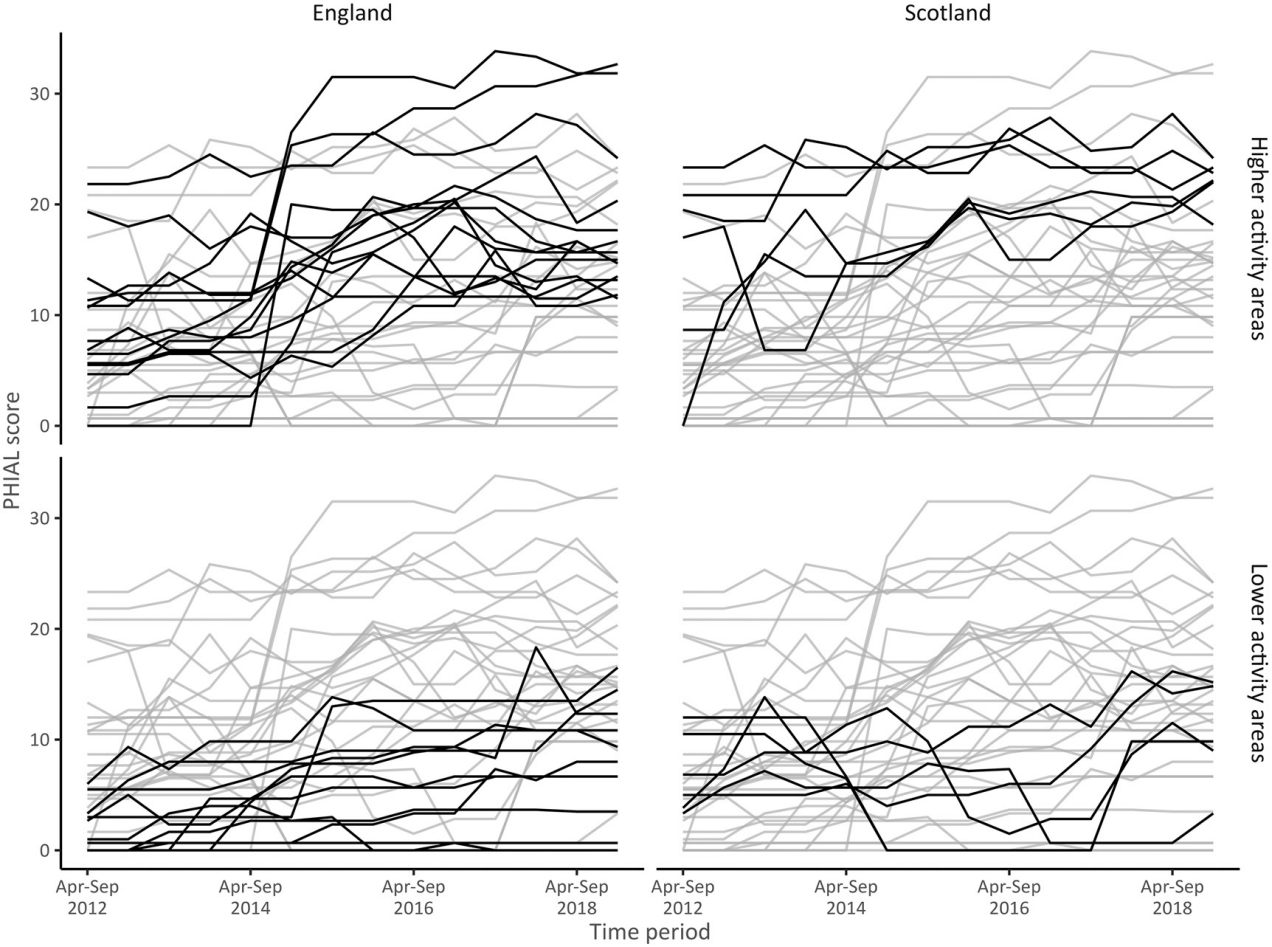
Temporal patterns of Public Health engagement In Alcohol Licensing (PHIAL) scores for 6-month periods from April 2012 to March 2019 for each of 39 participating local government areas.

Figure 1 shows the temporal patterns of PHT engagement in alcohol licensing as quantified by the PHIAL measure for each area. Temporal patterns between areas differed widely, with the highest PHIAL score an area had for any 6-month period being 35 out of the maximum possible 42.

Associations between 18-month average PHIAL scores (OSM Figure S3) and outcomes are shown in Table 1. Analyses indicate little evidence of correlations between average scores, lagged or unlagged, and health outcomes. Although 6-month lagged average scores were associated with higher public order offences (0.0105 (95% Confidence Interval (CI) 0.0027, 0.0183), 1-holdout resampling indicated that this depended on inclusion of one particular English area; exclusion reduced the effect estimate by 33% to 0.0007 (range 0.0007 to 0.0161; *p* > 0.10).

**Table 1:** Associations (per PHIAL unit exposure) of primary exposure metric (18 months average PHIAL score) and selected outcomes.

	18-month average Public Health Team Engagement Score					
	Unadjusted results			Adjusted results		
Outcome	Effectα	95%Confidence Interval	*P* value	Effectα	95%Confidence Interval	*P* value
**Effects on health outcomes**						
alcohol-related hospital admissions (narrow) *^β^*	0.0021	-0.0058,0.0099	0.605	0.0006	-0.0065,0.0078	0.866
acute alcohol-related hospital admissions *^β^*	0.0058	-0.0042, 0.0158	0.257	0.0033	-0.0058,0.0123	0.476
alcohol-related mortality	0.0012	-0.0018,0.0041	0.444	0.0016	-0.0015, 0.0047	0.315
alcohol-specific mortality	0.0040	-0.0030,0.0110	0.264	0.0035	-0.0032,0.0102	0.300
ambulance callouts*^β^*	0.0002	-0.0017,0.0022	0.808	0.0004	-0.0016, 0.0024	0.709
**Effects on crime outcomes**						
public order offences	**0.0354**	**0.0234, 0.0475**	**0.000**	0.0074	-0.0006,0.0153	0.068
sexual crimes	**0.0092**	**0.0035,0.0148**	**0.001**	-0.0007	-0.0055,0.0042	0.789
violent crimes	**0.0162**	**0.0107,0.0217**	**0.000**	0.0010	-0.0025,0.0044	0.574

	6-month lagged 18 months average Public Health Team Engagement Score					
	Unadjusted results			Adjusted results		
Outcome	Effect	95%Confidence Interval	*P* value	Effect	95%Confidence Interval	*P* value
**Effects on health outcomes**						
alcohol-related hospital admissions (narrow)*^β^*	0.0018	-0.0058,0.0094	0.643	0.0003	-0.0067,0.0073	0.935
acute alcohol-related hospital admissions*^β^*	0.0055	-0.0046,0.0155	0.285	0.0029	-0.0062,0.0120	0.534
alcohol-related mortality	-0.0002	-0.0031,0.0027	0.905	0.0004	-0.0027,0.0034	0.813
alcohol-specific mortality	0.0018	-0.0050,0.0085	0.611	0.0016	-0.0050,0.0082	0.640
ambulance callouts *^β^*	0.0000	-0.0019,0.0019	0.970	0.0007	-0.0014,0.0027	0.521
**Effects on crime outcomes**						
public order offences	**0.0380**	**0.0261,0.0499**	**<0.001**	**0.0105**	**0.0027,0.0183**	**0.008**
sexual crimes	**0.0076**	**0.0023,0.0130**	**0.005**	-0.0026	-0.0072, 0.0021	0.280
violent crimes	**0.0162**	**0.0108,0.0216**	**<0.001**	0.0003	-0.0031,0.0037	0.853

α Effect estimate (β) describes the change in outcome (^β^ per 100 events) with one unit change in 18-month average PHIAL score.

Associations between cumulative PHIAL score, both lagged and unlagged, and all outcomes are shown in Table 2. Statistically significant negative correlations were observed with alcohol-related mortality of -0.0004 (95%CI -0.0007,-0.0000; *p*∼0.049) and -0.0004 (95%CI -0.0008,-0.0001; *p*∼0.021) for unlagged and 6-month lagged cumulative PHIAL exposure, respectively. 1-Holdout resampling showed associations were non-significant for 33% of samples and significance relied on the inclusion of two specific areas (one in England and one in Scotland). A positive association with public order offences was observed (0.0012 (0.0004,0.021; *p*∼0.003) which was stable in holdout resampling.

**Table 2:** Associations (per PHIAL unit exposure) of cumulative public health team engagement score and selected outcomes.

	Cumulative Public Health Team Engagement Score					
	Unadjusted Results			Adjusted Results		
Outcome	Effectα	95%Confidence Interval	*P* value	Effectα	95%Confidence Interval	*P* value
**Effects on health outcomes**						
alcohol-related hospital admissions (narrow)*^β^*	0.0001	-0.0008,0.0011	0.817	0.0000	-0.0009,0.0008	0.956
acute alcohol-related hospital admissions*^β^*	0.0007	-0.0007,0.0020	0.323	0.0004	-0.0008,0.0016	0.537
alcohol-related mortality	-0.0003	-0.0007,0.0000	0.075	**-0.0004**	**-0.0007,-0.0000**	**0.049**
alcohol-specific mortality	-0.0003	-0.0011,0.0006	0.537	-0.0003	-0.0001,0.0005	0.445
ambulance callouts *^β^*	**-0.0003**	**-0.0005,-0.0000**	**0.019**	-0.0001	-0.0004,0.0001	0.187
**Effects on crime outcomes**						
public order offences	0.0000	-0.0013,0.0015	0.930	**0.0012**	**0.0004,0.0020**	**0.004**
sexual crimes	0.0003	-0.0003,0.0009	0.284	0.0001	-0.0004,0.0006	0.771
violent crimes	0.0001	-0.0005,0.0007	0.744	0.0002	-0.0001,0.0006	0.199

	6-month lagged cumulative Public Health Team Engagement Score					
	Unadjusted results			Adjusted results		
Outcome	Effect	95%CI	*P* value	Effect	95%CI	*P* value
**Effects on health outcomes**						
alcohol-related hospital admissions (narrow)*^β^*	0.0001	-0.0009,0.0011	0.860	-0.000	-0.0010,0.0009	0.931
acute alcohol-related hospital admissions*^β^*	0.0007	-0.0008,0.0021	0.354	0.0004	-0.0009,0.0017	0.563
alcohol-related mortality	**-0.0004**	**-0.0008,-0.0000**	**0.035**	**-0.0004**	**-0.0008,-0.0001**	**0.021**
alcohol-specific mortality	-0.0004	-0.0013,0.0005	0.381	-0.0004	-0.0012,0.0004	0.315
ambulance callouts*^β^*	**-0.0003**	**-0.0005,-0.0001**	**0.012**	-0.0002	-0.0004,0.0001	0.139
**Effects on crime outcomes**						
public order offences	-0.0004	-0.0018,0.0011	0.634	**0.0012**	**0.0004,0.0021**	**0.003**
sexual crimes	0.0002	-0.0004,0.0009	0.461	0.0001	-0.0005,0.0006	0.823
violent crimes	0.0001	-0.0007,0.0006	0.819	0.0002	-0.0001,0.0006	0.219

α Effect estimate (β) describes the change in outcome (^β^ per 100 events) with one unit change in cumulative PHIAL score.

Changes in the PHIAL measure were not correlated with any of the selected outcomes (Table 3), with the exception of a correlation between the unlagged change in PHIAL score and alcohol-related mortality (0.0050 [95%CI 0.0008,0.0091; 0.020], which was robust in 1-holdout resampling (range effect size 0.0044-0.0056; *p* ≤ 0.05).

**Table 3:** Associations (per PHIAL unit exposure) of changes in public health team engagement score and selected outcomes.

	Difference Public Health Team Engagement Score					
	Unadjusted results			Adjusted results		
Outcome	Effectα	95%Confidence Interval	*P* value	Effectα	95%Confidence Interval	*P* value
**Effects on health outcomes**						
alcohol-related hospital admissions (narrow) *^β^*	-0.0008	-0.0126,0.0110	0.894	0.0001	-0.0117,0.0119	0.990
acute alcohol-related hospital admissions *^β^*	-0.0028	-0.0284,0.0228	0.829	-0.0006	-0.0263,0.0250	0.961
alcohol-related mortality	**0.0063**	**0.0021,0.0105**	**0.003**	**0.0050**	**0.0008,0.0091**	**0.020**
alcohol-specific mortality	0.0058	-0.0039,0.0156	0.240	0.0055	-0.0042,0.0151	0.269
ambulance callouts *^β^*	0.0013	-0.0015,0.0041	0.348	0.0012	-0.0016,0.0040	0.409
**Effects on crime outcomes**						
public order offences	-0.0130	-0.031,0.0048	0.151	-0.0050	-0.0150,0.0051	0.333
sexual crimes	-0.0011	-0.0082,0.0061	0.772	0.0038	-0.0024,0.0100	0.231
violent crimes	-0.0004	-0.0112,0.0042	0.367	0.0018	-0.0028,0.0063	0.446

	Lagged difference Public Health Team Engagement Score					
	Unadjusted results			Adjusted results		
Outcome	Effect	95%CI	*P* value	Effect	95%CI	*P* value
**Effects on health outcomes**						
alcohol-related hospital admissions (narrow) *^β^*	0.0019	-0.0101,0.0138	0.761	0.0013	-0.0106,0.0133	0.829
acute alcohol-related hospital admissions *^β^*	0.0068	-0.0186,0.0322	0.600	0.0056	-0.0196,0.0308	0.663
alcohol-related mortality	-0.0000	-0.0043,0.0043	0.989	0.0011	-0.0033,0.0054	0.628
alcohol-specific mortality	0.0046	-0.0054,00146	0.369	0.0055	-0.0044,0.0155	0.277
ambulance callouts*^β^*	-0.0003	-0.0031,0.0025	0.838	0.0000	-0.0028,0.0028	0.986
**Effects on crime outcomes**						
public order offences	-0.0070	-0.0248,0.0108	0.442	-0.0094	-0.0195,0.0008	0.070
sexual crimes	0.0034	-0.0036,0.0104	0.344	0.0043	-0.0018,0.0104	0.168
violent crimes	-0.0005	-0.0081,0.0071	0.894	-0.0001	-0.0046,0.0044	0.981

α Effect estimate (β) describes the change in outcome (^β^ er 100 events) with one unit change in the change in PHIAL score.

Nation-specific analyses for 6-month lagged results are presented in OSM Table S4, and show negative associations for 18-month average PHIAL score and sexual crimes (-0.0088 [-0.0171, -0.0005]; *p* = 0.039) and for cumulative exposure and alcohol-specific mortality (-0.0011 [-0.0020,-0.0001]; *p* = 0.036) in Scotland, but not in England.

Additional *posthoc* analyses considered longer term effects, and modelled 12-month and 18-month lags (OSM Table S5), as well as considering associations between cumulative exposure to PHT activity over the full data collection period (7 years) and later outcomes, but results did not materially differ from the primary analyses.

## Discussion

This study aimed to assess associations between local PHT engagement with alcohol licensing and selected health and crime outcomes. The study provides little evidence that increased engagement had a measurable impact. A positive correlation of 6-month lagged 18-month average PHIAL scores, the primary exposure metric, with the incidence of public order offences was observed, but we consider this an artefact given (a) that this contradicts the direction of a plausible association, and (b) 1-holdout resampling indicated this association was reliant on the inclusion of a single area. This implausible association might possibly be related to issues of the recording of alcohol-related crimes and compliance with the Code of Practice in earlier years of the timeseries, discussed in detail in^9^. Similarly, negative associations were observed for alcohol-related mortality and cumulative PHIAL score in our secondary analysis, but these associations also relied on the inclusion of specific areas. Moreover, we hypothesize that if such an association were to exist, it would be more likely to show up with the alcohol-specific mortality measure in the timeframe of our study period. We observed negative associations with alcohol-specific mortality in Scotland however, as well as with sexual crimes, but not in England, which we interpret as chance findings given the small sample size in Scotland and absence of associations with related outcomes.

In summary therefore, our findings provide little evidence that the extent to which PHTs engage in diverse activities to influence alcohol premises licensing policies and decisions is associated with reductions in health harms or crimes linked to alcohol. There are several possible explanations for the largely null findings of this study. Although it may be that the activities of PHTs do not materially change local alcohol licensing policies and decisions at all, we have accumulated extensive qualitative data in the ExILEnS study suggesting that this is unlikely to be the case.^18^ This is also supported by other qualitative studies, including^19^, ^20^, ^21^ and by reviews of statements of licensing policy in Scotland.^22^ It may simply be that the extent of public health influence was not substantial enough to lead to changes in harms of a detectable magnitude. The alcohol licensing system is a long-standing highly legalistic local ‘centre’ of policymaking with cultures of evidence and practice that are very different from those of public health; for those seeking to change it, long-term engagement may be necessary to see substantial impact.^23^^,^^24^ It may also be therefore that public health activity takes longer to make an observable difference to harms, although additional analyses incorporating 12 and 18 months extended periods and cumulative PHT activity over a seven year period did not provide evidence to support this. In several areas, public health activity focused more on responsible retailing of alcohol rather than containing availability, and evidence supporting the effectiveness of the former in reducing harms is weak.^18^ Another, perhaps stronger, explanation may be that licensing policies and decisions can only currently have a very limited effect on alcohol harms within the context and constraints of the relatively permissive licensing systems in Scotland and England. Alcohol outlet density in the UK is high by international standards,^25^ and even the strongest licensing policies, where applications were routinely declined, could not legally reduce outlet numbers, but merely contain them at current levels. In fact, licence applications can continue to be granted, even in cumulative impact areas.^26^ Previous studies have shown a small positive effect of stronger licensing policies across a larger sample of local authorities in England,^8^^,^^10^^,^^27^ and in one London authority,^28^ but did not analyse the role of PHTs. Further, we analysed outcomes at the level of Local Government which might have been too large to detect more localised effects of public health actions. Previous research shows that effects can be measurable in small areas with good, hyper-local information on interventions, exposure and outcomes.^29^ Finally, because the process of influencing alcohol policies and implementing changes in alcohol environments sits within a complex system with many actors and widely varying local contexts, it is also possible that the linear exposure-response analyses aimed at obtaining average effects used here might not have been the most appropriate.

This study has several strengths. It was (to our knowledge) the first study to quantitatively examine the direct association between public health engagement in licensing and local health and crime outcomes. Secondly, the assessments of PHT activity were based on a novel semi-quantitative composite measure of the intensity of such activity (the PHIAL measure) that we developed prior to these analyses and was designed specifically with data from the areas in this study, and we *a priori* highlighted the primary and secondary exposure metrics. The study was based on a matched sample of areas of higher and lower engagement, but comparable with respect to other relevant characteristics. Furthermore, inferences were strengthened by the longitudinal nature of the data. Temporal patterns of primary exposure varied between and within areas over time, avoiding erroneous inferences resulting from positive or negative correlations between exposure and outcome observed in all areas across the time period. This is similarly a strength for the ‘change-in-PHIAL score’ metric, but not for cumulative exposure which increases over the study period and might be a contributing factor for the observed association with A&E attendances for alcohol.

An important limitation of this study is that the PHIAL exposure metric itself is not intuitively interpretable. Although the semiquantitative score enables the assessment of correlations between exposures and outcomes, the practical implication of observed associations is unclear. The PHIAL measure is also unlikely to have captured all possible public health approaches to engagement, due to recall limitations, and the scores generated are to some extent based on subjective judgment. The analyses would further have benefitted from additional information on alcohol licensing policies and environmental changes that, for example, may have affected alcohol availability and related harms in the areas, and which would have enabled modelling of complete hypothesized causal pathways. Our sample size was large considering the volume of primary data collection in the 39 areas, and was informed by statistical power calculations based on the direct effect of licensing decisions on alcohol-related hospital admissions and crimes based on previous research.^13^ Given the indirect effect PHT engagement in licensing would have on downstream health and crime outcomes we might expect smaller effects, if any, in which case our study would have benefitted from a larger sample size.

Although this quantitative evaluation provides little evidence of a direct impact of PHT engagement in licensing on health and crime outcomes, complementary qualitative evidence collected within the ExILEnS study indicates that PHTs provided valued input into alcohol licensing in ways which might reasonably be expected to facilitate reductions in alcohol harms.^18^ The mechanisms through which changes in availability may impact on alcohol harms remain poorly examined, and further research is needed to better understand exactly how changes in the alcohol retail environment, including temporal and physical availability, impact on alcohol consumption choices and patterns, and therefore, related harms.^30^ Nevertheless, the practical implication for public health of this research as a whole, is to realise that although PHTs can probably have some positive influence in slowly re-orienting local alcohol licensing to consider health outcomes and data, but that it is unrealistic to expect such efforts to translate directly into measurable improvements in health or crime in the 7 year timeframe of this study. For national policymakers, this implies that continued strengthening of licensing systems might be needed to achieve significant improvements in outcomes referenced in licensing objectives (preventing crime and disorder, and in Scotland, protecting and improving public health).

In conclusion, our findings provide little evidence that PHT engagement in licensing has a measurable downstream effect on health and crime in England or Scotland.

Whilst complementary qualitative evidence from the ExiLEns study suggests that allocating PHT resources to engaging in alcohol licensing is perceived as beneficial to achieving licensing objectives, this is not evidenced by measurable by measurable downstream reductions in crime and adverse health related to alcohol misuse, at least in the medium term.

## Contributors

These analyses were part of the larger ExILEnS project led by NF and conceived by NF, ME, FdV, CA, NS, LM, LB, and JN. AM, RP, NM, NF and ME recruited public health teams. NF, ME, FdV, CA, NS, LM, JN, CM,GC and MH contributed to the development of the exposure metric. AM, RP, NM,ROD and NF conducted exposure data collection, coded exposure data, and assigned exposure scores. CM, CF, PW obtained all outcome data and created the project data files. FdV led on the analyses, to which CM, CF and PW contributed. All authors contributed to the interpretation of the findings. FdV wrote the first draft of the manuscript and all authors commented on subsequent versions. All authors saw and approved the final manuscript.

## Data sharing statement

Data on alcohol-related hospital admissions and mortality for England can be obtained from PHE Local Alcohol Profiles (https://fingertips.phe.org.uk/profile/local-alcohol-profiles). Data on Scottish crimes were obtained from the Justice Analytical Services of the Scottish Government and English data were obtained from the Home Office and are open access at the Local Authority spatial aggregation (of crime location) level. The PHIAL measure will be published separately and can be obtained on request from the corresponding author.

## Declaration of interests

Colin Angus has received funding related to commissioned research from Systembolaget and Alko, the Swedish and Finnish government-owned alcohol retail monopolies. Other authors declare no competing interests.

## Appendix Supplementary materials

Image, application 1

Image, application 2
