# Supplementary material for: Impact of public health team engagement in alcohol licensing on health and crime outcomes in England and Scotland: A comparative timeseries study between 2012 and 2019
Source: Lancet Reg Health Eur. 2022 Jun 30;20:100450. doi: 10.1016/j.lanepe.2022.100450 (PMC9253894; doi:10.1016/j.lanepe.2022.100450)
Supplement: Supplementary file 1 [file mmc1.docx]

**Caption for supplementary material:** Online Supplement Materials
