# Supplementary material for: Impact of public health team engagement in alcohol licensing on health and crime outcomes in England and Scotland: A comparative timeseries study between 2012 and 2019
Source: Lancet Reg Health Eur. 2022 Jun 30;20:100450. doi: 10.1016/j.lanepe.2022.100450 (PMC9253894; doi:10.1016/j.lanepe.2022.100450)
Supplement: Supplementary file 2 [file mmc2.pdf]

Impact of public health team engagement in alcohol licensing on health and crime outcomes in England and Scotland: A comparative timeseries study between 2012-2019.

Authors: Frank de Vocht, PhD [1,2,3,], Cheryl McQuire, PhD[1,2], Claire Ferraro, MBBS[1], Philippa Williams, MSc[1], Madeleine Henney, MSc[4], Colin Angus, MSc[4,5], Matt Egan, PhD[6], Andrea Mohan, PhD[7], Richard Purves, PhD[8], Nason Maani, PhD[6,9], Niamh Shortt, PhD[10,5], Laura Mahon, MA[5,8,11], Gemma Crompton,MSC[11], Rachel O'Donnell[8], James Nicholls[8], Linda Bauld, PhD[12,5], Niamh Fitzgerald, PhD[8,5*]

[1] Population Health Sciences, Bristol Medical School, University of Bristol, UK

[2] NIHR School for Public Health Research, UK

[3] NIHR Applied Research Collaboration West

[4] School of Health and Related Research, University of Sheffield

[5] SPECTRUM Consortium, UK

[6] Department of Public Health, Environments and Society, London School of Hygiene & Tropical Medicine

[7] School of Health Sciences, University of Dundee

[8] Institute for Social Marketing & Health, University of Stirling

[9] Boston University School of Public Health, USA

[10] School of GeoSciences, University of Edinburgh

[11] Alcohol Focus Scotland

[12] Usher Institute, University of Edinburgh

ONLINE SUPPLEMENTARY INFORMATION

*** Corresponding author:** Professor Niamh Fitzgerald, Institute for Social Marketing and Health, University of Stirling, Stirling, FK9 4LA. Email: niamh.fitzgerald@stir.ac.uk

Funding: The ExILEnS project is funded by the NIHR Public Health Research Programme (project number 15/129/11). The views expressed are those of the authors and not necessarily those of the NHS, the NIHR or the Department of Health.

Table S1: Profile of participating public health team areas

Local Authority	Level of Government & Urban/Rural Classification for England*	Region	Average (2012-2018) population density per km^2	Average (2012-2018) Index of Multiple Deprivation	Average (2012-2018) alcohol-related hospital admissions (narrow)	Average (2012-2018) public order offenses
**1**	Unitary (4: Urban with city and town)	North East and Yorkshire	1300-1400	29	2500-2600	1000-1100
**2**	Unitary* (4: Urban with city and town)	London and South East	600-700	22	1400-1500	600-700
**3**	Unitary (4: Urban with city and town)	North East and Yorkshire	400-500	40	2500-2600	600-700
**4**	Unitary (6: Urban with major conurbation)	London and South East	800-900	32	15200-15300	600-700
**5**	Unitary (4: Urban with city and town)	London and South East	800-900	27	5200-5300	500-600
**6**	Lower tier* (3: Urban with significant rural)	London and South East	100-200	8	300-400	300-400
**7**	Unitary (6: Urban with major conurbation)	North West	500-600	27	2000-2100	1300-1400
**8**	Lower tier (4: Urban with city and town)	South West	300-400	15	100-200	100-200
**9**	Unitary (4: Urban with city and town)	North West	400-500	42	4000-4100	700-800
**10**	Lower tier (4: Urban with city and town)	North East and Yorkshire	1000-1100	27	900-1000	1400-1500
**11**	Unitary (1: mainly rural)	South West	600-700	23	100-200	2000-2100
**12**	Unitary (6: Urban with major conurbation)	London and South East	300-400	15	5400-5500	400-500

**13**	Lower Tier (4: Urban with city and town)	North West	300-400	28	900-1000	400-500
**14**	Lower tier (2: Largely rural)	London and South East	100-200	25	100-200	300-400
**15**	Unitary (4: Urban with city and town)	East	400-500	25	4200-4300	500-600
**16**	Lower tier (3: Urban with significant rural)	North West	200-300	23	200-300	500-600
**17**	Unitary (6: Urban with major conurbation)	North West	300-400	42	1700-1800	600-700
**18**	Lower tier (4: Urban with city and town)	South East	200-300	22	2000-2100	300-400
**19**	Unitary (6: Urban with major conurbation)	North East	1100-1200	30	2000-2100	1300-1400
**20**	Lower tier (3: Urban with significant rural)	North West	100-200	31	800-900	200-300
**21**	Lower tier (4: Urban with city and town)	South East	300-400	9	500-600	300-400
**22**	Unitary (6: Urban with major conurbation)	London	900-1000	35	14700-14800	500-600
**23**	Lower tier (2: Largely Rural)	South West	300-400	16	100-200	400-500
**24**	Lower tier (4: Urban with city and town)	East Midlands	200-300	28	2700-2800	300-400
**25**	Lower tier (3: Urban with significant rural)	South East	200-300	23	300-400	300-400
**26**	Lower tier (3: Urban with significant rural)	South East	100-200	11	300-400	200-300

**27**	Lower tier (4: Urban with city and town)	East	300-400	23	1600-1700	400-500
**28**	1&2: Large and Other Urban areas	Scotland West	400-500	30	500-600	400-500
**29**		Scotland West	100-200	11	500-600	300-400
**30**		Scotland East	100-200	17	0-100	400-500
**31**		Scotland West	9000-9100	35	3400-3500	3200-3300
**32**		Scotland NE	900-1000	15	1200-1300	800-900
**33**		Scotland East	600-700	29	2400-2500	500-600
**34**		Scotland East	100-200	21	400-500	700-800
**35**		Scotland West	300-400	28	100-200	600-700
**36**		Scotland East	500-600	21	200-300	1500-1600
**37**		Scotland West	1700-1800	23	100-200	1200-1300
**38**		Scotland	1800-1900	28	700-800	1500-1600
**39**		Scotland NE	200-300	19	10-30	300-400

* Many parts of England have both a county council (or upper tier authority) and a district council (or lower tier authority). County councils run public services such as education, libraries, roads and social care, whilst district councils are responsible for matters such as waste, environment and housing. In some other areas, instead of upper/lower tier authorities, a single (unitary) council is responsible for all these services. Urban-rural classification for England is available at local authority level [30], but not for Scotland, where a breakdown of urban-rural geography specific to each local authority would identify the area in question.

Table S2: Exposure activities included in the PHIAL Measure

Activity Category	Activity Category Definition	Activity Types Graded in the PHIAL Measure
1. Staffing for PHT activity to influence local alcohol licensing	Staffing of PHT activity to influence local alcohol licensing.	1.1 Senior Leadership<br>1.2 Staff Continuity
2. Reviewing alcohol licensing applications:	Engaging in an activity or process to decide whether to take action in relation to individual alcohol licensing applications.	2.1 New licence applications/licence variations (other than 2.2)<br>2.2 Reviewing or monitoring applications or decisions relating to temporary increases in availability<br>2.3 Monitoring responses to applications
3. Influencing & responding to individual licence applications	Engaging in any activity to influence the submission, type, content or outcome of alcohol licensing applications (excluding that covered elsewhere).	3.1 Influencing or Preventing Applications Prior to Submission<br>3.2 Shaping Submitted Applications Prior to Decision<br>3.3 Making representations or objections<br>3.4 Involvement in reviews of premises licences<br>3.5 Involvement in appeals to decisions resulting from 3.3
4. Use of Routine or Bespoke Data on Alcohol Licensing and Alcohol-Related Harms.	Collection, collation, analysis, or other use of data (other than specified in 2.3 above or 6.1 below) to inform, or use in support of, PHT activity to influence local alcohol licensing	4.1 Collation or Analysis of Existing Data<br>4.2 Establishing New or Expanded Data Collection Processes
5. Influencing local stakeholders or licensing policy	Any activity to influence licensing policy or people, or other stakeholders (other than the public).	5.1 Contributing to the development of Licensing Policy<br>5.2 Influencing or Collaborating with Local government Licensing Team and associated services<br>5.3 Informing or Influencing Elected Representatives responsible for licensing decisions.<br>5.4 Involvement in formal or statutory multi-agency licensing groups.<br>5.5 Collaboration with statutory bodies with legal responsibilities in relation to alcohol licensing.
6. Engagement or involvement of the public	Any activity to engage or involve the public in relation to alcohol licensing including the use of media.	6.1 Contact, collaboration or initiatives with members of the public or community groups regarding alcohol licensing<br>6.2 Media publicity

Table S3. Overview of selected study outcomes and sources of data

	Selected outcome	Source	
		**England**	**Scotland**
1	alcohol-related hospital admissions (narrow)	Public Health England (now UKHSA) Local Alcohol Profiles	Information Services Division (ISD) Scotland
2	acute alcohol-related hospital admissions	Public Health England (now UKHSA) Local Alcohol Profiles	Information Services Division (ISD) Scotland
3	alcohol-related mortality	Office for National Statistics	National Records of Scotland (NRS)
4	alcohol-specific mortality	Office for National Statistics	National Records of Scotland (NRS)
5	ambulance callouts	Ambulance services	Scottish Ambulance Service
6	public order offences	Office for National Statistics	Scottish Government
7	sexual crimes	Office for National Statistics	Scottish Government
8	violent crimes	Office for National Statistics	Scottish Government

1: Estimates of the number of alcohol-related hospital admissions have been calculated by applying alcohol-attributable fractions (AAFs) to Hospital Episode Statistics data.[1]

2: Alcohol-related hospital admissions excluding chronic conditions.[1]

3: Alcohol-specific deaths plus diseases where only a proportion of the deaths were caused by alcohol (such as cancers of the mouth, oesophagus and liver).[1]

4: Conditions where each death is a direct consequence of alcohol misuse. The definition is primarily based on chronic (longer-term) conditions associated with continued misuse of alcohol and, to a lesser extent, acute (immediate) conditions.[1]

5: Ambulance callouts includes all ambulance callouts where an ambulance was dispatched to the scene, and these were geocoded to the Local Authority based on address of incident. Scottish ambulance data were obtained directly from the Scottish Ambulance Service, while English ambulance data were obtained from the East of England Ambulance Service, London Ambulance Service, North East Ambulance Service, North West Ambulance Service, South Central Ambulance Service, South East Coast Ambulance Service, South Western Ambulance Service, Yorkshire Ambulance Service, East Midlands Ambulance Service, and West Midlands Ambulance Service through individual data specification. Data for one English area was not available for 2012 and 2013 because of a change in the computer system and for another 6 English areas data for 2013 were missing because of an error in providing the data.

6: Number of recorded incidents. The absolute numbers for crimes from both countries are not directly comparable because included crimes and definitions do not directly correspond in England and Scotland.

7: Number of recorded incidents. The absolute numbers for crimes from both countries are not directly comparable because included crimes and definitions do not directly correspond in England and Scotland.

8: Number of recorded incidents. The absolute numbers for crimes from both countries are not directly comparable because included crimes and definitions do not directly correspond in England and Scotland.

[1] NHS Digital. Statistics on Alcohol, England 2020. February 4, 2020. https://digital.nhs.uk/data-and-information/publications/statistical/statistics-on-alcohol/2020

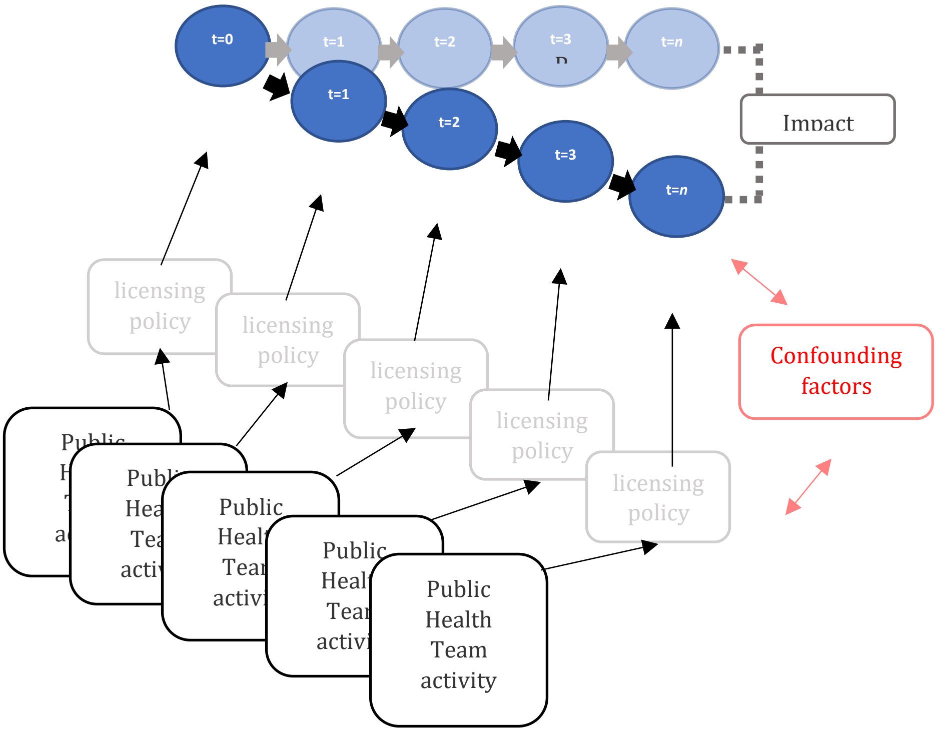

Figure S1. Snare model evaluating impact of public health team activity (through influencing licensing policies). Note that cumulative PHT activity at t=3 is t=0+t=1+t=2.

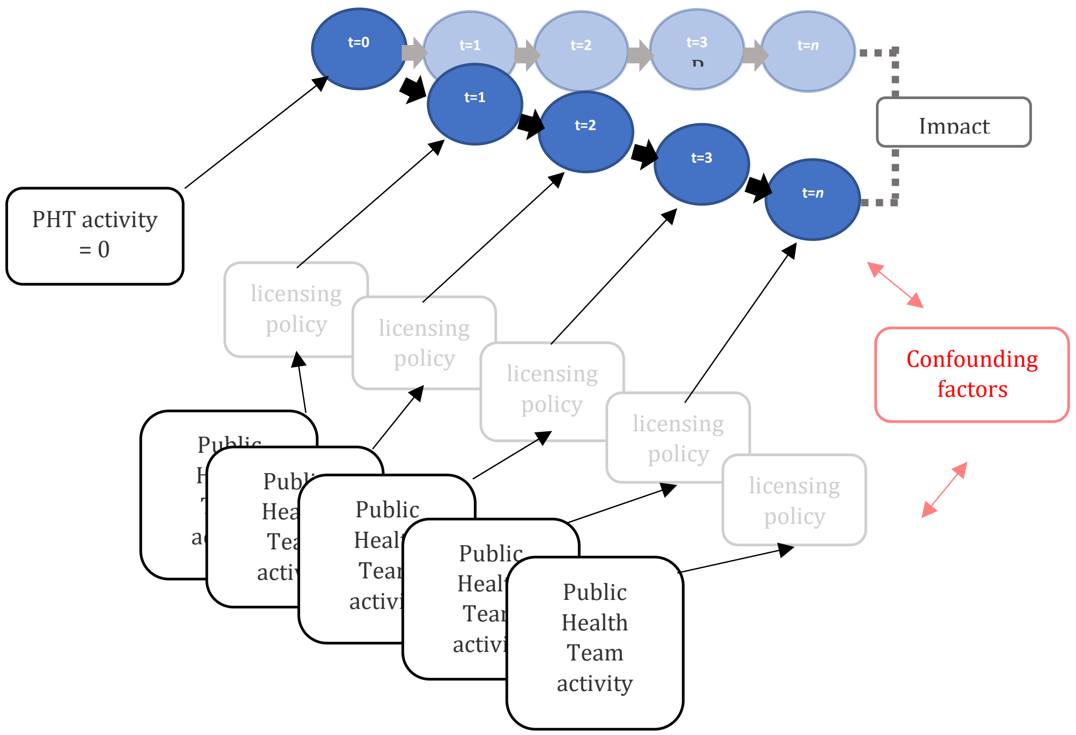

Figure S2. Snare model evaluating impact of public health team activity (through influencing licensing policies) on outcomes in the subsequent 6-month period. Note that cumulative PHT activity at t=3 is t=0+t=1+t=2; impacting on outcome t=4.

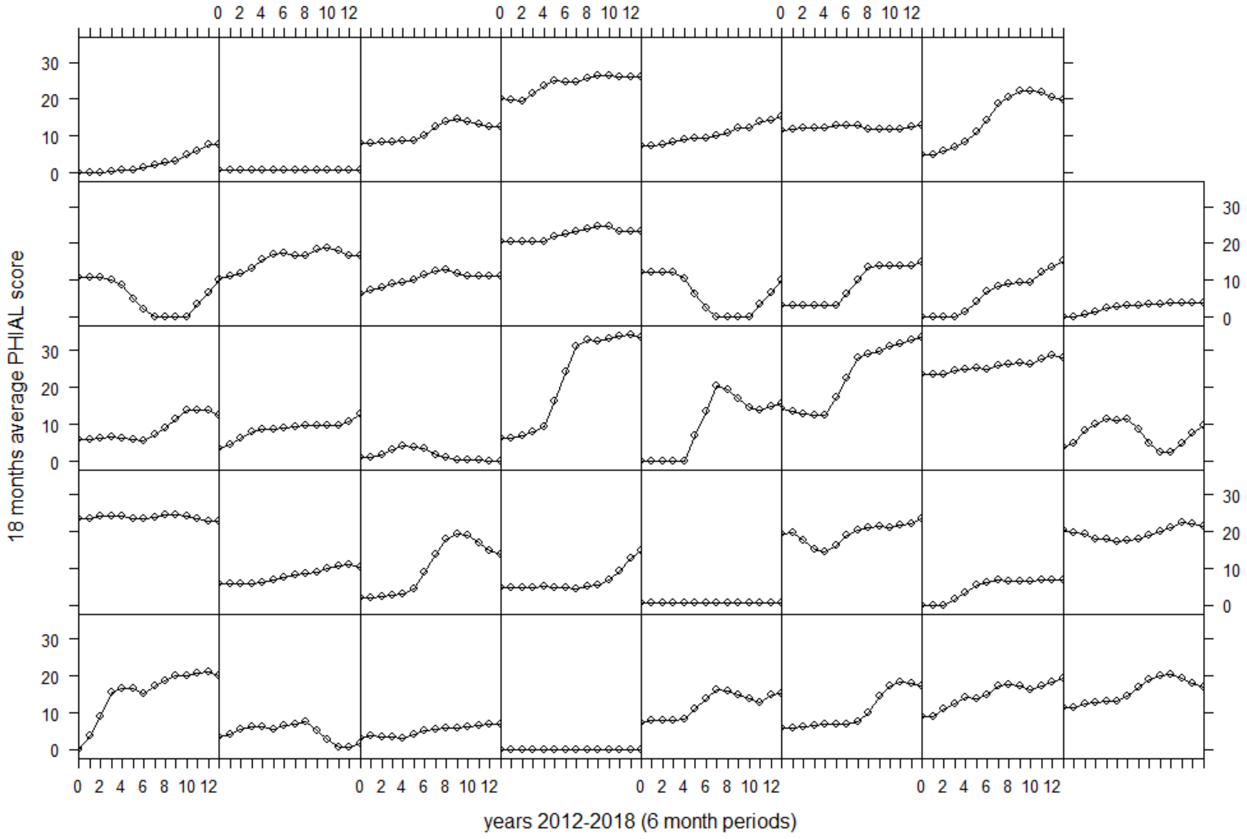

Figure S3. Graphical overview of the primary exposure metric, 18-month average PHIAL score, for each of the 39 participating areas.

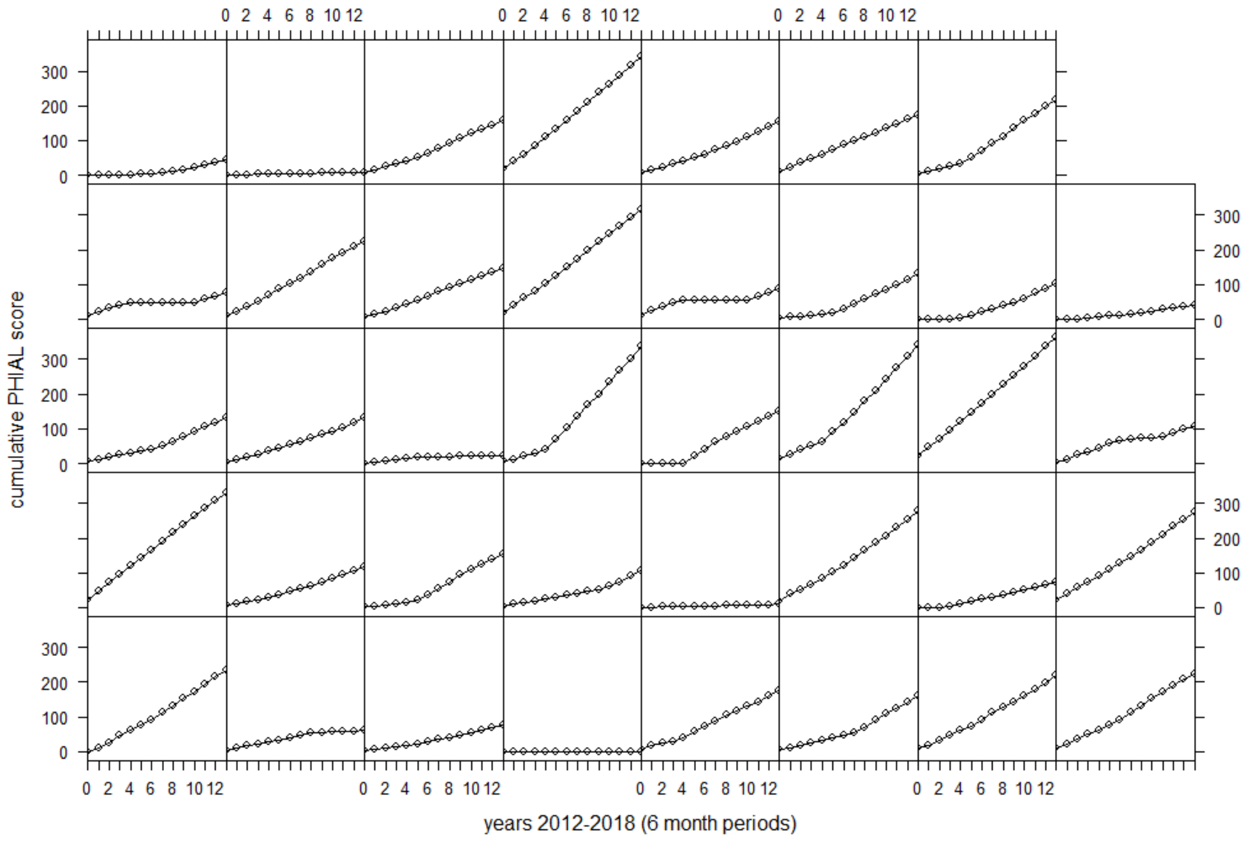

Figure S4. Graphical overview of the cumulative PHIAL score, one of the secondary exposure metrics, for each of the 39 participating areas.

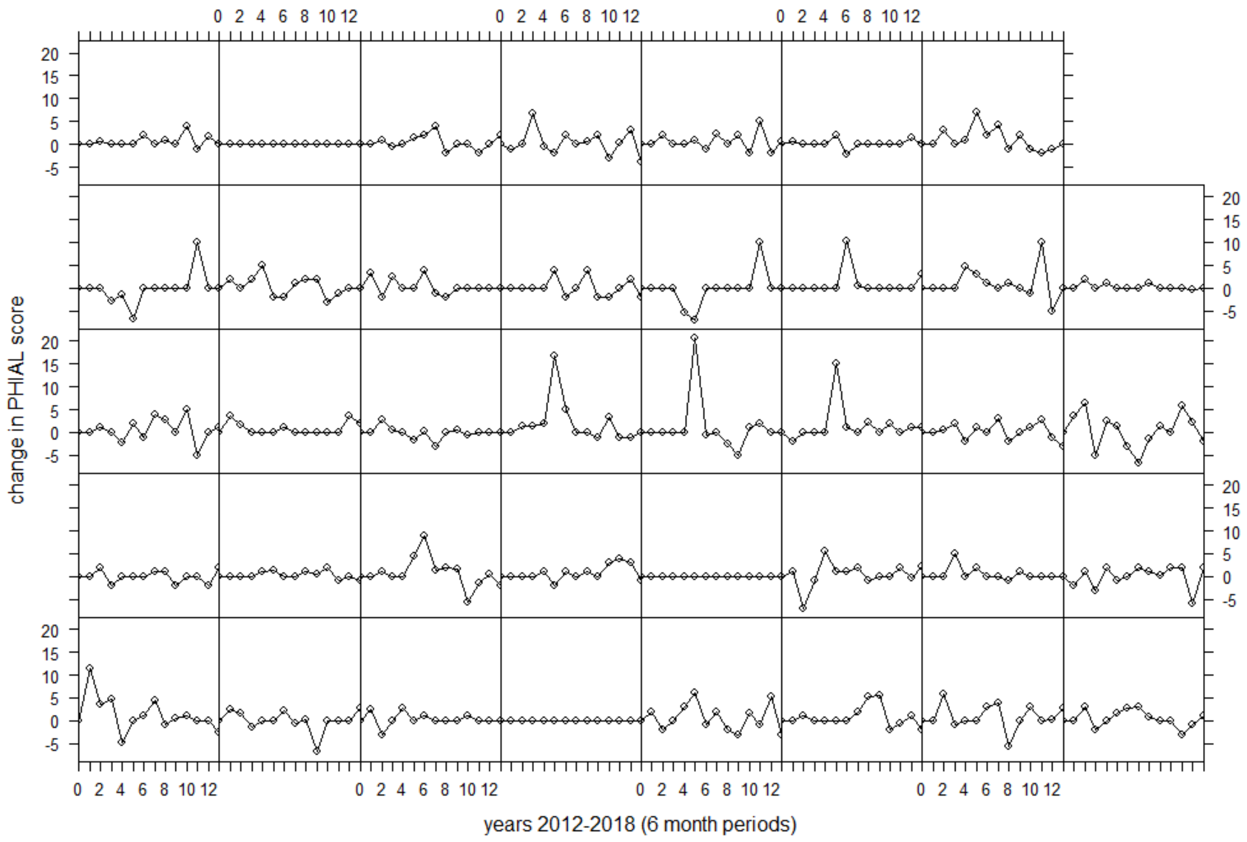

Figure S5. Graphical overview of the change in PHIAL score at each 6 month period compared to the previous period, one of the secondary exposure metrics, for each of the 39 participating areas.

Table S4. Country-specific associations (per PHIAL unit exposure) of 6-months lagged 18 months average PHIAL score and cumulative PHIAL score and selected outcomes.

**6-month lagged 18-months average PHIAL score[α]**						
	**ENGLAND**			**SCOTLAND**		
**Outcome**	**Effect[β]**	**95%Confidence Interval**	**P value**	**Effect[β]**	**95%Confidence Interval**	**P value**
**Effects on health outcomes**						
alcohol-related hospital admissions (narrow) [γ]	0.0006	-0.0100,0.0112	0.910	0.0043	-0.0027,0.0113	0.232
acute alcohol-related hospital admissions [γ]	0.0038	-0.0090,0.0166	0.560	0.0017	-0.0124,0.0157	0.818
alcohol-related mortality	0.0013	-0.0032,0.0059	0.561	-0.0004	-0.0042,0.0034	0.823
alcohol-specific mortality	0.0044	-0.0057,0.0147	0.390	-0.0040	-0.0113,0.0033	0.280
ambulance callouts [γ]	0.0014	-0.0013,0.0041	0.303	-0.0011	-0.0042,0.0021	0.509
**Effects on crime outcomes**						
public order offenses	**0.0101**	**0.0005,0.0198**	**0.040**	**0.0161**	**0.0043,0.0279**	**0.008**
sexual crimes	0.0013	-0.0040,0.0067	0.627	**-0.0088**	**-0.0171,-0.0005**	**0.039**
violent crimes	0.0008	-0.0036,0.0053	0.717	-0.0015	-0.0057,0.0027	0.479
**6-month lagged Cumulative PHIAL score***						
**Effects on health outcomes**	**ENGLAND**			**SCOTLAND**		
alcohol-related hospital admissions (narrow) [γ]	-0.0001	-0.0014,0.0011	0.857	0.0004	-0.0007,0.0015	0.511
acute alcohol-related hospital admissions [γ]	0.0004	-0.0014,0.0022	0.664	0.0001	-0.0022,0.0024	0.950
alcohol-related mortality	-0.0004	-0.0009,0.0001	0.105	-0.0004	-0.0009,0.0001	0.125
alcohol-specific mortality	-0.0000	-0.0012,0.0012	0.961	**-0.0011**	**-0.0020,-0.0001**	**0.036**
ambulance callouts [γ]	-0.0003	-0.0006,0.0001	0.110	-0.0001	-0.0006,0.0003	0.498
**Effects on crime outcomes**						
public order offenses	**0.0014**	**0.0004,0.0024**	**0.007**	0.0008	-0.0005,.0020	0.233
sexual crimes	0.0003	-0.0003,0.0009	0.315	-0.0003	-0.0013,0.0006	0.490
violent crimes	0.0004	-0.0001,0.0009	0.122	-0.0003	-0.0007,0.0002	0.279

[α]*: modelled using the same statistical models as used for main analyses, with the exception of country-specific time trends and removal of 'local alcohol action' in Scottish models*

[β] *Effect estimate (β) describes the change in outcome ([γ] er 100 events) with one unit change in PHIAL exposure metric*

Table S5. Associations (per PHIAL unit exposure) of primary exposure metric (18 months average PHIAL score) and selected outcomes with extended (12 and 18-month) exposure lag periods.

	18-months average PHIAL score					
	12-month lagged			18-month lagged		
**Outcome**	**Effect [α]**	**95%Confidence Interval**	**P value**	**Effect [α]**	**95%Confidence Interval**	**P value**
**Effects on health outcomes**						
alcohol-related hospital admissions (narrow) [β]	-0.0000	-0.0070,0.0069	0.989	-0.0003	-0.0074,0.0067	0.929
acute alcohol-related hospital admissions [β]	0.0023	-0.0070,0.0117	0.623	0.0019	-0.0075,0.0113	0.695
alcohol-related mortality	-0.0000	-0.0031,0.0030	0.966	-0.0008	-0.0038,0.0023	0.631
alcohol-specific mortality	0.0010	-0.0056,0.0076	0.771	0.0008	-0.0058,0.0075	0.808
ambulance callouts [β]	0.0003	-0.0017,0.0024	0.748	-0.0001	-0.0022,0.0019	0.901
**Effects on crime outcomes**						
public order offenses	**0.0141**	**0.0065,0.0217**	**0.000**	**0.0169**	**0.0094,0.0244**	**0.000**
sexual crimes	-0.0034	-0.0078,0.0011	0.138	-0.0038	-0.0080,0.0006	0.094
violent crimes	0.0001	-0.0033,0.0035	0.958	-0.0001	-0.0035,0.0032	0.934

[α] *Effect estimate (β) describes the change in outcome ([βγ] er 100 events) with one unit change in PHIAL exposure metric*
